# dl-Alaninium iodide

**DOI:** 10.1107/S1600536812022003

**Published:** 2012-05-23

**Authors:** Kevin Lamberts, Ulli Englert

**Affiliations:** a Institut für Anorganische Chemie, RWTH Aachen University, Landoltweg 1, 52074 Aachen, Germany

## Abstract

The crystal structure of dl-alanine hydro­iodide (1-carb­oxy­ethanaminium iodide), C_3_H_8_NO_2_^+^·I^−^, is that of an organic salt consisting of *N*-protonated cations and iodide anions. The compound features homochiral helices of N—H⋯O hydrogen-bonded cations in the [010] direction; neighbouring chains are related by crystallographic inversion centers and hence show opposite chirality. The iodide counter-anions act as hydrogen-bond acceptors towards H atoms of the ammonium and carb­oxy groups, and cross-link the chains along [100]. Thus, an overall two-dimensional network is formed in the *ab* plane. No short contacts occur between iodide anions.

## Related literature

For related structures of l-alanine hydro­chloride, see: Di Blasio *et al.* (1977 ▶), d-alanine alaninium bromide, see: Fischer (2006 ▶), l-alanine hydro­chloride monohydrate, see: Yamada *et al.* (2008 ▶) and dl-alanine hydro­chloride, see: Trotter (1962 ▶).
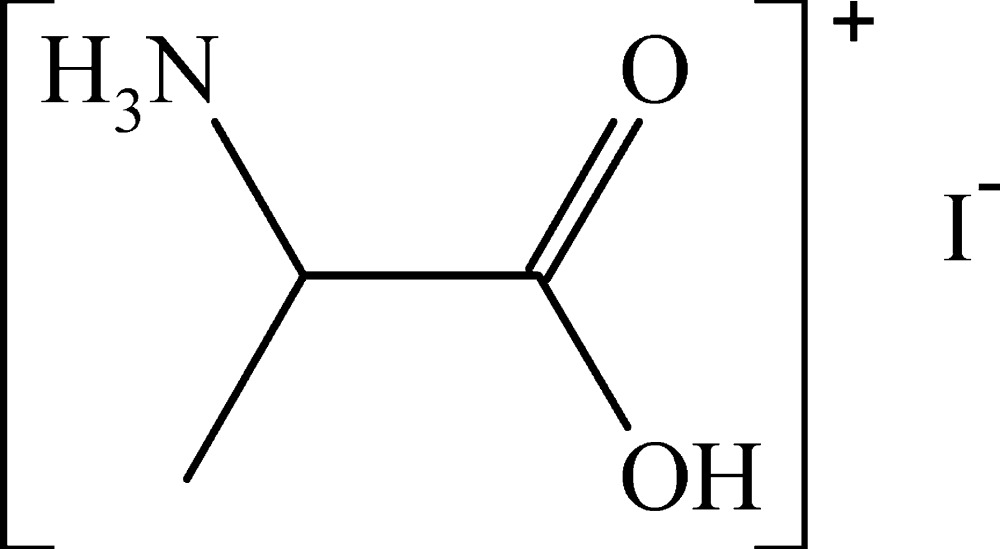


## Experimental

### 

#### Crystal data

C_3_H_8_NO_2_^+^·I^−^
*M* *_r_* = 217.00
Monoclinic, 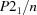

*a* = 7.6975 (11) Å
*b* = 5.7776 (8) Å
*c* = 16.034 (2) Å
β = 98.999 (2)°
*V* = 704.30 (17) Å^3^
*Z* = 4
Mo *K*α radiation
μ = 4.46 mm^−1^
*T* = 100 K
0.30 × 0.11 × 0.05 mm

#### Data collection

Bruker D8 goniometer with SMART APEX CCD detector
Absorption correction: multi-scan (*SADABS*; Bruker, 2001 ▶) *T*_min_ = 0.348, *T*_max_ = 0.808
10196 measured reflections
2109 independent reflections
1887 reflections with *I* > 2σ(*I*)
*R*_int_ = 0.055

#### Refinement

*R*[*F* ^2^ > 2σ(*F* ^2^)] = 0.026
*wR*(*F* ^2^) = 0.062
*S* = 1.05
2109 reflections
77 parameters
3 restraints
H atoms treated by a mixture of independent and constrained refinement
Δρ_max_ = 1.17 e Å^−3^
Δρ_min_ = −1.87 e Å^−3^

### 

Data collection: *SMART* (Bruker, 2001 ▶); cell refinement: *SAINT* (Bruker, 2001 ▶); data reduction: *SAINT*; program(s) used to solve structure: *SHELXS97* (Sheldrick, 2008 ▶); program(s) used to refine structure: *SHELXL97* (Sheldrick, 2008 ▶); molecular graphics: *PLATON* (Spek, 2009 ▶); software used to prepare material for publication: *publCIF* (Westrip, 2010 ▶).

## Supplementary Material

Crystal structure: contains datablock(s) I, global. DOI: 10.1107/S1600536812022003/nk2163sup1.cif

Structure factors: contains datablock(s) I. DOI: 10.1107/S1600536812022003/nk2163Isup2.hkl

Supplementary material file. DOI: 10.1107/S1600536812022003/nk2163Isup3.cml

Additional supplementary materials:  crystallographic information; 3D view; checkCIF report

## Figures and Tables

**Table 1:** Hydrogen-bond geometry (Å, °)

*D*—H⋯*A*	*D*—H	H⋯*A*	*D*⋯*A*	*D*—H⋯*A*
O1—H1⋯I1	0.79 (4)	2.61 (4)	3.391 (2)	171 (3)
N1—H1*A*⋯O2^i^	0.87 (3)	2.05 (3)	2.861 (3)	155 (3)
N1—H1*B*⋯I1^ii^	0.88 (3)	2.71 (3)	3.557 (2)	163 (3)
N1—H1*C*⋯I1^iii^	0.87 (3)	2.80 (3)	3.580 (2)	150 (3)

Symmetry codes: (i) 
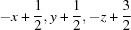
; (ii) 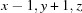; (iii) 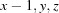.

**Fig. 1.**
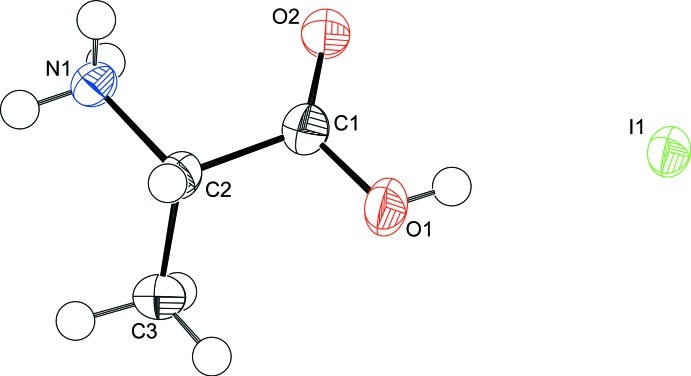
: PLATON (Spek, 2009) plot with displacement ellipsoids scaled to 80% probability, H atoms shown as spheres with arbitrary radii.

**Fig. 2.**
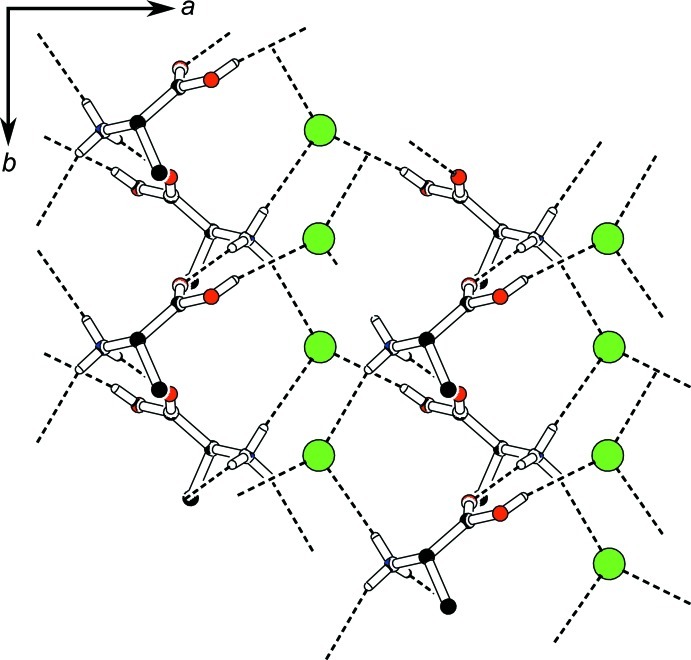
: Representation of the C-face, showing a top view of the two-dimensional layer built by hydrogen bonds (Spek, 2009).

**Fig. 3.**
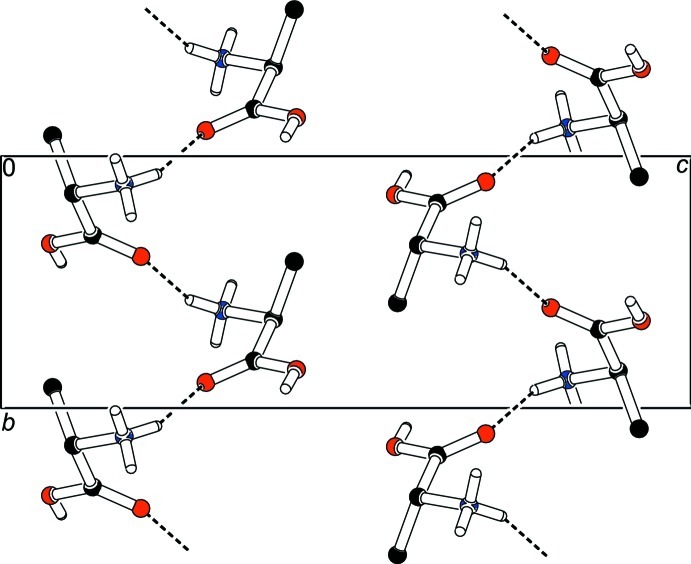
: View on the A-face; homochiral helices extend along [010] (Spek, 2009).

## supplementary crystallographic information

### Comment

Our attempt to synthesize a coordination compound from manganese(II)iodide and the racemic α-amino acid DL-alanine failed and unexpectedly led to the formation of the title compound.

The structure of this organic salt consists of one protonated alanine cation and one iodide anion in the asymmetric unit (Fig. 1); the compound crystallizes in the monoclinic space group *P*2_1_*/n*.

All H atoms bonded to electronegative partners find an acceptor in suitable geometry (Table 1), thus forming the maximum number of classical hydrogen bonds. These interactions give rise to double layers (Fig. 2), with the iodide acting as acceptor for one donor from the carboxylic acid OH and two from the ammonium group; the halide adopts a trigonal-planar geometry with respect to these hydrogen bonds. A fourth hydrogen bond is formed between the remaining proton in the ammonium group and a neighbouring carboxylic acid O atom, forming a helical structure along the *b*-axis (Fig. 3). Each helix is homochiral, but the centrosymmetry of the space group implies the presence of left- and right-handed helices related by crystallographic inversion.

### Experimental

MnI_2_ 4H_2_O (0.2 mmol, 74 mg) and DL-alanine (0.4 mmol, 36 mg) were dissolved in 5 ml H_2_O/MeOH (1:1) and were left in an open flask at room temperature. After slow evaporation of the solvent a yellow oil remained which was placed in a desiccator. Colorless needles of DL-alanine hydroiodide formed after several weeks.

### Refinement

Hydrogen atoms bonded to carbon were included as riding in standard geometry with C—H = 1.00 Å for the methine and C—H = 0.98 Å for the methyl C atom. Coordinates of the hydrogen atoms in the ammonium and in the carboxylic acid groups were refined freely, with the N—H distances restrained to equal length. For all H atoms, *U*_iso_(H) was constrained to 1.2 *U*_eq_ of the non-H reference atom.

### Crystal data

**Table:**

C_3_H_8_NO_2_^+^·I^−^	*F*(000) = 408
*M**_r_* = 217.00	*D*_x_ = 2.047 Mg m^−^^3^
Monoclinic, *P*2_1_/*n*	Mo *K*α radiation, λ = 0.71073 Å
Hall symbol: -P 2yn	Cell parameters from 3131 reflections
*a* = 7.6975 (11) Å	θ = 2.6–30.3°
*b* = 5.7776 (8) Å	µ = 4.46 mm^−^^1^
*c* = 16.034 (2) Å	*T* = 100 K
β = 98.999 (2)°	Needle, colourless
*V* = 704.30 (17) Å^3^	0.30 × 0.11 × 0.05 mm
*Z* = 4	

### Data collection

**Table:**

Bruker D8 goniometer with SMART APEX CCD detector diffractometer	2109 independent reflections
Radiation source: INCOATEC microsource	1887 reflections with *I* > 2σ(*I*)
Multilayer optics monochromator	*R*_int_ = 0.055
ω scans	θ_max_ = 30.7°, θ_min_ = 2.6°
Absorption correction: multi-scan (*SADABS*; Bruker, 2001)	*h* = −10→11
*T*_min_ = 0.348, *T*_max_ = 0.808	*k* = −8→8
10196 measured reflections	*l* = −22→22

### Refinement

**Table:**

Refinement on *F*^2^	Primary atom site location: structure-invariant direct methods
Least-squares matrix: full	Secondary atom site location: difference Fourier map
*R*[*F*^2^ > 2σ(*F*^2^)] = 0.026	Hydrogen site location: inferred from neighbouring sites
*wR*(*F*^2^) = 0.062	H atoms treated by a mixture of independent and constrained refinement
*S* = 1.05	*w* = 1/[σ^2^(*F*_o_^2^) + (0.020*P*)^2^ + 0.4*P*] where *P* = (*F*_o_^2^ + 2*F*_c_^2^)/3
2109 reflections	(Δ/σ)_max_ = 0.001
77 parameters	Δρ_max_ = 1.17 e Å^−^^3^
3 restraints	Δρ_min_ = −1.87 e Å^−^^3^

### Special details

**Table:**

Geometry. All e.s.d.'s (except the e.s.d. in the dihedral angle between two l.s. planes) are estimated using the full covariance matrix. The cell e.s.d.'s are taken into account individually in the estimation of e.s.d.'s in distances, angles and torsion angles; correlations between e.s.d.'s in cell parameters are only used when they are defined by crystal symmetry. An approximate (isotropic) treatment of cell e.s.d.'s is used for estimating e.s.d.'s involving l.s. planes.
Refinement. Refinement of *F*^2^ against ALL reflections. The weighted *R*-factor *wR* and goodness of fit *S* are based on *F*^2^, conventional *R*-factors *R* are based on *F*, with *F* set to zero for negative *F*^2^. The threshold expression of *F*^2^ > σ(*F*^2^) is used only for calculating *R*-factors(gt) *etc*. and is not relevant to the choice of reflections for refinement. *R*-factors based on *F*^2^ are statistically about twice as large as those based on *F*, and *R*- factors based on ALL data will be even larger.

### Fractional atomic coordinates and isotropic or equivalent isotropic displacement parameters (Å^2^)

**Table:**

	*x*	*y*	*z*	*U*_iso_*/*U*_eq_	
I1	0.78595 (2)	0.38517 (3)	0.868059 (10)	0.01622 (7)	
O1	0.4337 (3)	0.6529 (4)	0.92827 (13)	0.0248 (5)	
H1	0.508 (5)	0.577 (6)	0.913 (2)	0.030*	
O2	0.2836 (3)	0.6020 (3)	0.79725 (13)	0.0193 (4)	
C1	0.3013 (3)	0.6870 (5)	0.86701 (16)	0.0162 (5)	
C2	0.1684 (3)	0.8526 (5)	0.89481 (17)	0.0158 (5)	
H2	0.1169	0.7800	0.9420	0.019*	
C3	0.2528 (4)	1.0818 (5)	0.9252 (2)	0.0249 (6)	
H3A	0.1619	1.1888	0.9380	0.030*	
H3B	0.3120	1.1483	0.8809	0.030*	
H3C	0.3390	1.0558	0.9761	0.030*	
N1	0.0254 (3)	0.8877 (4)	0.82138 (14)	0.0143 (4)	
H1A	0.060 (4)	0.928 (5)	0.7744 (16)	0.017*	
H1B	−0.052 (4)	0.988 (5)	0.834 (2)	0.017*	
H1C	−0.036 (4)	0.761 (4)	0.811 (2)	0.017*	

### Atomic displacement parameters (Å^2^)

**Table:**

	*U*^11^	*U*^22^	*U*^33^	*U*^12^	*U*^13^	*U*^23^
I1	0.01442 (11)	0.01740 (11)	0.01628 (10)	0.00331 (6)	0.00066 (7)	0.00025 (6)
O1	0.0196 (10)	0.0337 (12)	0.0190 (10)	0.0129 (9)	−0.0036 (8)	−0.0060 (9)
O2	0.0183 (10)	0.0227 (11)	0.0159 (9)	0.0043 (7)	0.0000 (8)	−0.0040 (7)
C1	0.0153 (12)	0.0167 (13)	0.0164 (12)	0.0011 (9)	0.0021 (9)	0.0011 (9)
C2	0.0146 (12)	0.0185 (13)	0.0136 (11)	0.0032 (9)	−0.0005 (9)	−0.0007 (9)
C3	0.0220 (14)	0.0223 (14)	0.0272 (15)	0.0040 (11)	−0.0059 (12)	−0.0102 (12)
N1	0.0146 (11)	0.0154 (11)	0.0119 (10)	0.0010 (8)	−0.0006 (8)	0.0005 (8)

### Geometric parameters (Å, º)

**Table:**

O1—C1	1.315 (3)	C3—H3A	0.98
O1—H1	0.79 (4)	C3—H3B	0.98
O2—C1	1.210 (3)	C3—H3C	0.98
C1—C2	1.517 (4)	N1—H1A	0.87 (2)
C2—N1	1.495 (3)	N1—H1B	0.88 (2)
C2—C3	1.521 (4)	N1—H1C	0.87 (2)
C2—H2	1.00		
			
C1—O1—H1	111 (3)	C2—C3—H3B	109.5
O2—C1—O1	126.3 (3)	H3A—C3—H3B	109.5
O2—C1—C2	123.0 (2)	C2—C3—H3C	109.5
O1—C1—C2	110.8 (2)	H3A—C3—H3C	109.5
N1—C2—C1	107.5 (2)	H3B—C3—H3C	109.5
N1—C2—C3	111.1 (2)	C2—N1—H1A	115 (2)
C1—C2—C3	111.7 (2)	C2—N1—H1B	110 (2)
N1—C2—H2	108.8	H1A—N1—H1B	110 (3)
C1—C2—H2	108.8	C2—N1—H1C	110 (2)
C3—C2—H2	108.8	H1A—N1—H1C	107 (3)
C2—C3—H3A	109.5	H1B—N1—H1C	103 (3)

### Hydrogen-bond geometry (Å, º)

**Table:**

*D*—H···*A*	*D*—H	H···*A*	*D*···*A*	*D*—H···*A*
O1—H1···I1	0.79 (4)	2.61 (4)	3.391 (2)	171 (3)
N1—H1*A*···O2^i^	0.87 (3)	2.05 (3)	2.861 (3)	155 (3)
N1—H1*B*···I1^ii^	0.88 (3)	2.71 (3)	3.557 (2)	163 (3)
N1—H1*C*···I1^iii^	0.87 (3)	2.80 (3)	3.580 (2)	150 (3)

Symmetry codes: (i) −*x*+1/2, *y*+1/2, −*z*+3/2; (ii) *x*−1, *y*+1, *z*; (iii) *x*−1, *y*, *z*.
